# Health Indicators as Measures of Individual Health Status and Their Public Perspectives: Cross-sectional Survey Study

**DOI:** 10.2196/38099

**Published:** 2022-06-21

**Authors:** Temiloluwa Sokoya, Yuchun Zhou, Sebastian Diaz, Timothy Law, Lina Himawan, Francisca Lekey, Lu Shi, Ronald W Gimbel, Xia Jing

**Affiliations:** 1 Department of Behavioral and Environmental Health College of Health Sciences Jackson State University Jackson, MS United States; 2 Gladys W and David H Patton College of Education Ohio University Athens, OH United States; 3 College of Medicine Northeast Ohio Medical University Rootstown, OH United States; 4 Ohio Musculoskeletal and Neurological Institute Ohio University Athens, OH United States; 5 Department of Psychology College of Arts and Sciences Ohio University Athens, OH United States; 6 College of Health Sciences and Professions Ohio University Athens, OH United States; 7 Department of Public Health Sciences College of Behavioral, Social, and Health Sciences Clemson University Clemson, SC United States

**Keywords:** health status measurement, individual health indicators, public perspectives, surveys and questionnaires

## Abstract

**Background:**

Disease status (eg, cancer stage) has been used in routine clinical practice to determine more accurate treatment plans. Health-related indicators, such as mortality, morbidity, and population group life expectancy, have also been used. However, few studies have specifically focused on the comprehensive and objective measures of individual health status.

**Objective:**

The aim of this study was to analyze the perspectives of the public toward 29 health indicators obtained from a literature review to provide evidence for further prioritization of the indicators. The difference between health status and disease status should be considered.

**Methods:**

This study used a cross-sectional design. Online surveys were administered through Ohio University, ResearchMatch, and Clemson University, resulting in three samples. Participants aged 18 years or older rated the importance of the 29 health indicators. The rating results were aggregated and analyzed as follows (in each case, the dependent variables were the individual survey responses): (1) to determine the agreement among the three samples regarding the importance of each indicator, where the independent variables (IVs) were the three samples; (2) to examine the mean differences between the retained indicators with agreement across the three samples, where the IVs were the identified indicators; and (3) to rank the groups of indicators into various levels after grouping the indicators with no mean differences, where the IVs were the groups of indicators.

**Results:**

In total, 1153 valid responses were analyzed. Descriptive statistics revealed that the top five–rated indicators were drug or substance abuse, smoking or tobacco use, alcohol abuse, major depression, and diet and nutrition. Among the 29 health indicators, the three samples agreed upon the importance of 13 indicators. Inferential statistical analysis indicated that some of the 13 indicators held equal importance. Therefore, the 13 indicators were categorized by rank into seven levels: level 1 included blood sugar level and immunization and vaccination; level 2 included LDL cholesterol; level 3 included HDL cholesterol, blood triglycerides, cancer screening detection, and total cholesterol; level 4 included health literacy rate; level 5 included personal care needs and air quality index greater than 100; level 6 included self-rated health status and HIV testing; and level 7 included the supply of dentists. Levels 1 to 3 were rated significantly higher than levels 4 to 7.

**Conclusions:**

This study provides a baseline for prioritizing 29 health indicators, which can be used by electronic health record or personal health record system designers or developers to determine what can be included in the systems to capture an individual’s health status. Currently, self-rated health status is the predominantly used health indicator. Additionally, this study provides a foundation for tracking and measuring preventive health care services more accurately and for developing an individual health status index.

## Introduction

Disease status, such as cancer stage, has frequently been used in routine clinical practice to determine more accurate treatment plans. Health-related indicators, such as mortality, morbidity, and life expectancy for a given population group, have also been used. Few studies, however, have focused on more comprehensive and objective measures of an individual’s health status. Self-rated health status has previously been identified as a reliable indicator of an individual’s overall health status [1,2], but this is subjective and the rating criteria are unclear. Although there is research on health indicators used for the measurement of care quality [3], as well as social and behavioral measures in electronic health record (EHR) systems [4,5], more comprehensive and objective health indicators of an individual’s health status are lacking. These must be developed and used to measure health status more accurately, objectively, and consistently, as well as to evaluate the outcomes of preventive medicine services [1,6]. As the health care paradigm shifts from treatment to prevention [7,8], the accurate, objective, and convenient measurement of preventive services and their long-term outcomes becomes an urgent and growing need.

Individual health status refers to a person’s overall physical, mental, and social well-being, as well as freedom from illness or injury. In contrast, individual disease status refers to a person’s physical or mental symptoms with or without diagnosis [9]. Accurate individual health status measures can guide customized preventive medicine services and lifestyle suggestions and be applied to population health programs by aggregating an individual’s health data into meaningful groups. Chronic diseases are increasingly costly to both patients and society, and most can be prevented or delayed via preventive medicine services. These services need to be provided in a routine and consistent manner [7,8], thus maximizing the potential to control health care costs.

The Institute of Medicine reviewed social and behavioral domain measures, as seen in EHR systems [4,5]. They identified 17 domains, and these measurements were used as a foundation for the Office of the National Coordinator for Health Information Technology’s Meaningful Use of EHRs reporting requirements [4,5]. In 2015, the Centers for Disease Control and Prevention’s National Center for Health Statistics released 15 selected health indicators based on the National Health Interview Survey [10]. Other research [1,2,6,11] also considered health indicators, although none focused on comprehensive, objective measures of an individual’s health status.

Although preventive medicine has been recognized for its critical role in health care, such services are not provided consistently to the majority of the American population [12]. Because chronic diseases represent a large portion of health care expenditures, it is critical to prevent or delay the onset of chronic diseases via preventive services [13]. The tracking of health indicators has been reported to help policy makers note changes needed in coverage and to influence policy decisions [14]. Such tracking also enables governments to better allocate health resources [14]. Nevertheless, accurate measurements of preventive services are inadequate or lacking.

We conducted a literature review of existing health indicators [1,2,6,11,12,15-17]. We consolidated the described health indicators and determined that 29 health indicators could be used to measure an individual’s health. We then examined four commercial EHR systems used in rural, primary care, and ambulatory settings to explore the availability and presentation of these indicators as a pilot study. None of these systems were found to capture *all* of the indicators [9], but each system provided data on some available health indicators, and all four systems had preventive medicine panels. The pilot study indicated that no established group of health indicators existed for individuals, nor were indicators incorporated or used consistently and routinely across different EHR systems. Therefore, there is at least a need to provide more evidence for these health indicators. This would constitute what should be included among the individual health indicators used by EHR systems and whether these indicators can be prioritized based on their importance. Additionally, we recognize that these health indicators have much broader potential use beyond incorporating them into EHR [9].

This study aimed to examine public perspectives on the importance of 29 health indicators to inform their relative perceived priority. This would, for example, allow the separation of the health indicators into core and secondary sets, which could be incorporated into the EHR or similar systems [18]. Such health indicators could capture an individual’s health status, thus informing and enabling preventive services to make them more accurate, consistent, and convenient without overburdening providers’ data collection workload. These public perspectives could also provide a foundation for developing an individual health index, which could be used to stratify healthy populations into subgroups based on the corresponding study requirements. There is no established list or ranking of health indicators according to importance, nor is there a well-established methodology to develop such a list. Therefore, we attempted to use public perspective surveys as a starting point in this study. We plan to validate the results quantitatively through longitudinal health record analysis in a future study. The assumption is that an individual’s perception of the importance of each health indicator may be associated with their conscious or unconscious behaviors, which would ultimately affect health outcomes. This paper focuses on the public perspectives, the approach, the results, and the analysis of the results.

## Methods

### General Study Design

**Figure 1:**
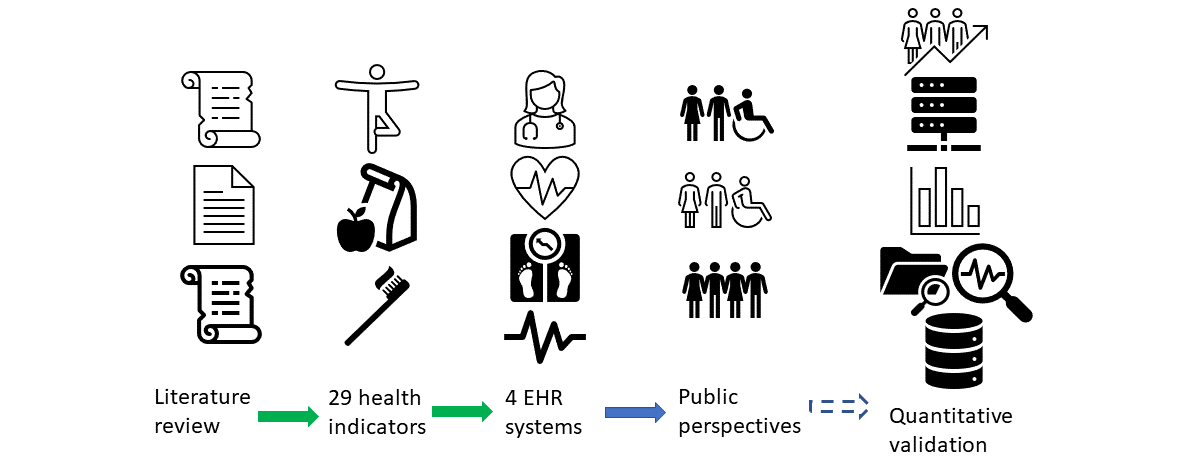
The overall design of the project; public perspectives are the focus of this paper. The three sections connected via green arrows have been completed, and the far-right section is for future work. EHR: electronic health record.

The overall design of this project is illustrated in Figure 1 to provide context for this paper. The work reported here focuses on public perspectives, the methods used, and the results. The first three steps in Figure 1 have been completed and published [9,18]. The section on the far-right side of the figure, regarding quantitative validation, illustrates directions we plan to explore in future studies.

### Data Collection

An online survey (Multimedia Appendix 1) was administered through Ohio University in the summer of 2017, through ResearchMatch [19] in the summer of 2018, and through Clemson University in the summer of 2020, providing three samples. The inclusion criterion for participation in the survey was being 18 years of age or older. The participants were allowed to share the survey’s URL link, and all respondents acknowledged informed consent.

The survey included seven demographic questions and rating items related to the importance of the 29 health indicators. Definitions of these health indicators were provided within the survey (Multimedia Appendix 2). In the survey, the 29 health indicators were separated into five subscales [1,2,17]:

1. Health risks and behaviors, with eight indicators: alcohol abuse, BMI, diet and nutrition, drug or substance abuse, family history of cancer, physical inactivity, smoking or tobacco use, and sun protection.
2. Health care, with three indicators: immunization and vaccination, insurance coverage, and personal care needs.
3. Health care provider supply, with three indicators: cancer screening detection, hypertension screening, and HIV testing.
4. Blood tests in physical exams, with five indicators: blood sugar level, blood triglycerides, high-density lipoprotein (HDL) cholesterol, low-density lipoprotein (LDL) cholesterol, and total cholesterol.
5. Other health indicators, with 10 indicators: self-rated health status, high school diploma, air quality index greater than 100, supply of dentists, engagement in life, health literacy rate, major depression, having a sense of purpose in one’s life, race and ethnicity, and being unemployed.

After removing invalid data, the final sample yielded 362 responses from Ohio University, 694 from ResearchMatch, and 97 from Clemson University (Multimedia Appendix 3). Survey items used by Ohio University and Clemson University were rated on a scale of 0 to 10, where 0 refers to “not at all important” and 10 refers to “extremely important.” Survey items in the ResearchMatch sample were measured using a scale of 0 to 100, where 0 refers to “not at all important” and 100 refers to “extremely important.” Therefore, as part of the data cleaning process, the data from ResearchMatch were converted to a scale of 0 to 10 (Multimedia Appendix 4 contains the codebook). In the Ohio University survey, there were five health indicators—blood sugar, blood triglycerides, HDL, LDL, and total cholesterol—for which a scale of 0 to 11, instead of 0 to 10, was used. Due to this error, data for these five indicators were removed from the Ohio University data set. As a result, the total sample size of these five indicators was 791 respondents, whereas the total sample size of the other indicators was 1153 respondents.

The internal reliability of the survey instruments was calculated for the overall set and the three institutional subsets using Cronbach α [20].

### Data Analytic Strategies

Data analyses included rating the 29 health indicators based on their perceived importance. After aggregating data from the three samples with descriptive statistics, a three-step analysis was conducted. The first step of the analysis involved determining whether the three samples had unanimous agreement on the importance of each indicator. A one-way analysis of variance (ANOVA) with a post hoc test was conducted using SPSS software (version 27; IBM Corp) for each indicator to examine any group mean difference, where the independent variables were the three samples and the dependent variables were the individual survey responses. A Levene test was used to test the homogeneity of variance for each indicator before running an ANOVA. The indicators with no group mean differences across samples were retained for the following analysis step.

The second step of the analysis examined the mean differences between the retained indicators via a one-way ANOVA, where the independent variables were the identified indicators and the dependent variables were the individual survey responses. Any indicators with no significant mean differences were grouped together because they could not be ranked.

The third step of the analysis ranked the groups of indicators into various levels after grouping the indicators with no mean differences. A one-way ANOVA with a post hoc test was conducted to examine the mean differences between the levels of indicators, where the independent variables were the levels of indicators and the dependent variables were the individual survey responses. Any significant mean difference between any two levels of indicators indicated the ranking order of the two levels.

### Ethics Approval

This study was approved by the Institutional Review Boards of Ohio University (17-X-142) and Clemson University (IRB2019-441).

## Results

### The Overall Layout of the Findings

**Figure 2:**
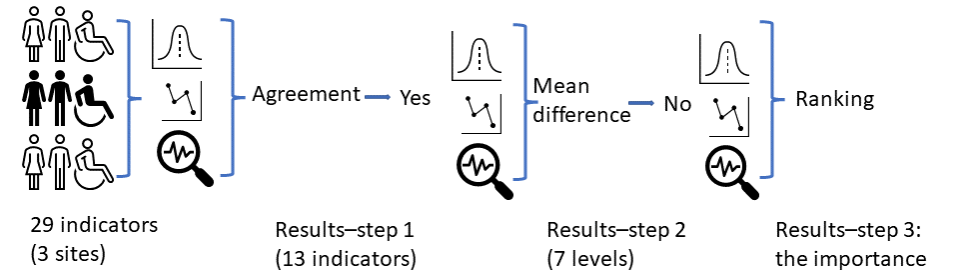
The primary analytic strategies and overall results of each step.

The primary purpose of this study was to identify the public perspectives on the importance of the 29 selected health indicators, whether they agreed with each other, and, if so, what were the importance rankings of the health indicators that agreed with each other. Figure 2 summarizes the analytic strategies and the main results of each step. The following paragraphs elaborate on the detailed results for each step.

### Results of Descriptive Statistics

Descriptive statistics for the 29 health indicators are reported in Table 1. The descriptive statistics for the demographic information for all respondents are reported in Multimedia Appendix 5. Descriptive analyses show that drug and substance abuse, smoking and tobacco use, alcohol abuse, major depression, and diet and nutrition were found to be the five most important health indicators, as rated by the study participants. Additionally, race and ethnicity, possession of a high school diploma, engagement in life, unemployment status, and sun protection were the five least important health indicators. Self-rated health status, the most-used health indicator to assess an individual’s health status, was ranked in the 20th position.

**Table 1:** Descriptive statistics for all 29 health indicators.

Health indicators	ResearchMatch (n=694)		Ohio University (n=362)		Clemson University (n=97)		Total (N=1153)	
	Score^a^, mean (SD)	n (%)	Score^a^, mean (SD)	n (%)	Score^a^, mean (SD)	n (%)	Score^a^, mean (SD)	n (%)
Drug or substance abuse	8.75 (1.5)	694 (100)	8.13 (1.96)	362 (100)	8.36 (1.87)	97 (100)	8.53 (1.71)	1153 (100)
Smoking and tobacco use	8.8 (1.52)	694 (100)	8.02 (2.06)	362 (100)	8.18 (1.84)	97 (100)	8.5 (1.77)	1153 (100)
Alcohol abuse	8.34 (1.71)	694 (100)	7.56 (2.03)	362 (100)	8.06 (1.64)	97 (100)	8.07 (1.84)	1153 (100)
Major depression	8.1 (1.6)	685 (98.7)	7.79 (1.94)	362 (100)	8.03 (1.57)	97 (100)	7.99 (1.72)	1144 (99.2)
Diet and nutrition	8.01 (1.58)	694 (100)	7.8 (1.93)	362 (100)	8.36 (1.65)	97 (100)	7.97 (1.71)	1153 (100)
Blood sugar level	7.76 (1.63)	694 (100)	N/A^b^	N/A	7.59 (1.75)	97 (100)	7.74 (1.65)	791 (68.6)
Physical inactivity	7.9 (1.68)	694 (100)	7.41 (2.13)	362 (100)	7.68 (1.77)	97 (100)	7.73 (1.85)	1153 (100)
Immunization and vaccination	7.49 (2.12)	694 (100)	7.67 (2.3)	362 (100)	7.72 (2.4)	97 (100)	7.57 (2.2)	1153 (100)
Hypertension screening	7.59 (1.91)	694 (100)	7.17 (2.29)	362 (100)	7.42 (2.03)	97 (100)	7.45 (2.05)	1153 (100)
LDL^c^ cholesterol	7.43 (1.85)	694 (100)	N/A	N/A	7.56 (1.91)	97 (100)	7.45 (1.86)	791 (68.6)
Blood triglycerides	7.32 (1.78)	694 (100)	N/A	N/A	7.34 (1.95)	97 (100)	7.32 (1.80)	791 (68.6)
HDL^d^ cholesterol	7.31 (1.83)	694 (100)	N/A	N/A	7.43 (1.91)	97 (100)	7.32 (1.84)	791 (68.6)
Having a sense of purpose in one’s life	7.59 (1.94)	685 (98.7)	6.67 (2.53)	362 (100)	7.88 (1.93)	97 (100)	7.32 (2.19)	1144 (99.2)
Cancer screening detection	7.22 (2.06)	694 (100)	7.26 (2.3)	362 (100)	7.49 (2.09)	97 (100)	7.25 (2.14)	1153 (100)
Total cholesterol	7.2 (2.02)	694 (100)	N/A	N/A	7.6 (1.85)	97 (100)	7.25 (2.00)	791 (68.6)
Health literacy rate	6.99 (2.02)	685 (98.7)	7.06 (2.26)	362 (100)	7.34 (2.01)	97 (100)	7.04 (2.10)	1144 (99.2)
Personal care needs	6.82 (2.08)	694 (100)	7.01 (2.3)	362 (100)	7.21 (2.1)	97 (100)	6.91 (2.16)	1153 (100)
Air quality index >100	6.74 (1.92)	685 (98.7)	6.76 (2.13)	362 (100)	6.89 (1.93)	97 (100)	6.76 (1.99)	1144 (99.2)
Family history of cancer	6.98 (2.06)	694 (100)	6.37 (2.24)	362 (100)	6.25 (1.98)	97 (100)	6.73 (2.13)	1153 (100)
Self-rated health status	6.63 (2.2)	694 (100)	6.62 (2.15)	362 (100)	6.92 (1.89)	97 (100)	6.65 (2.16)	1153 (100)
HIV testing	6.62 (2.36)	694 (100)	6.62 (2.64)	362 (100)	6.84 (2.37)	97 (100)	6.64 (2.45)	1153 (100)
Insurance coverage	6.4 (2.88)	694 (100)	6.79 (2.91)	362 (100)	7.26 (2.51)	97 (100)	6.6 (2.87)	1153 (100)
BMI	6.86 (2.28)	694 (100)	5.8 (2.54)	362 (100)	6.64 (2.45)	97 (100)	6.51 (2.42)	1153 (100)
Supply of dentists	6.53 (2.02)	685 (98.7)	6.34 (2.26)	362 (100)	6.04 (1.99)	97 (100)	6.43 (2.10)	1144 (99.2)
Sun protection	6.63 (2)	694 (100)	5.73 (2.3)	362 (100)	5.54 (2.18)	97 (100)	6.25 (2.16)	1153 (100)
Unemployed individual	6.07 (2.34)	685 (98.7)	5.52 (2.68)	362 (100)	6.2 (2.69)	97 (100)	5.91 (2.49)	1144 (99.2)
Engagement in life	6.38 (2.18)	685 (98.7)	4.82 (2.87)	362 (100)	6.4 (2.33)	97 (100)	5.89 (2.54)	1144 (99.2)
High school diploma as a health indicator	5.02 (2.57)	694 (100)	6.04 (3.07)	362 (100)	5.56 (2.75)	97 (100)	5.38 (2.79)	1153 (100)
Race and ethnicity	5.28 (2.53)	685 (98.7)	4.32 (2.76)	362 (100)	5.02 (2.85)	97 (100)	4.96 (2.67)	1144 (99.2)

^a^Items were rated on a scale of 0 to 10, where 0 refers to “not at all important” and 10 refers to “extremely important.”

^b^N/A: not applicable; data for this indicator were removed from the Ohio University data set because a scale of 0 to 11 vs 0 to 10 was used.

^c^LDL: low-density lipoprotein.

^d^HDL: high-density lipoprotein.

### Results of Inferential Statistics

A Levene test was conducted to test the homogeneity of variance for each indicator before running an ANOVA. This resulted in nine health indicators with homogenous variance: blood sugar level, HDL cholesterol, LDL cholesterol, total cholesterol, immunization and vaccination, insurance coverage, cancer screening detection, air quality index greater than 100, and self-rated health status (Multimedia Appendix 6). A total of 20 health indicators were found to have heterogeneous variance. These included the following indicators: blood triglycerides, alcohol abuse, BMI, diet and nutrition, drug or substance abuse, family history of cancer, physical inactivity, smoking and tobacco use, sun protection, personal care needs, hypertension screening, HIV testing, high school diploma as a health indicator, supply of dentists, engagement in life, health literacy rate, major depression, having a sense of purpose in one’s life, race and ethnicity, and unemployment (Multimedia Appendix 7).

For the nine indicators with homogeneous variance, a one-way ANOVA was used. A one-way ANOVA Welch test was used for the 20 indicators with heterogeneous variance. As a result, 13 indicators were found to have no statistically significant mean differences among the three samples. This indicates that survey participants generally agreed on the relative level of importance of these indicators (Table 2). The 13 indicators were blood sugar level, blood triglycerides, HDL cholesterol, LDL cholesterol, total cholesterol, personal care needs, HIV testing, self-rated health status, supply of dentists, health literacy rate, immunization and vaccination, cancer screening detection, and air quality index greater than 100. The means and SDs of their ratings are presented in Table 2. These 13 indicators were retained for the second step of the analysis. Significant mean differences were found among the other 16 indicators, which indicates that survey participants disagreed on their level of importance (Multimedia Appendix 8 contains the post hoc results).

In the second step of the analysis, a one-way ANOVA was run for the 13 retained indicators, where the independent variables were the 13 indicators and the dependent variables were the individual survey responses. The indicators with no mean differences were grouped into the same level (Table 3) because they were rated as equally important and could not be ranked within a level. As a result, seven levels were formed (Table 3). Level 1 to level 7 rankings were organized based on the mean importance of the health indicators from high to low within and between levels. Level 1 included blood sugar level and immunization and vaccination; level 2 included LDL cholesterol; level 3 included HDL cholesterol, blood triglycerides, cancer screening detection, and total cholesterol; level 4 included health literacy rate; level 5 included personal care needs and air quality index greater than 100; level 6 included self-rated health status and HIV testing; and level 7 included the supply of dentists.

In the third step of the analysis, a one-way ANOVA was used to rank the seven levels of indicators, where the independent variables were the seven levels and the dependent variables were the individual survey responses. There were seven indicators in levels 1 to 3: blood sugar level, immunization and vaccination, LDL cholesterol, HDL cholesterol, blood triglycerides, cancer screening detection, and total cholesterol. These indicators were found to be significantly more important to the survey participants than the six indicators ranked in levels 4 to 7: health literacy rate, personal care needs, air quality index greater than 100, self-rated health status, HIV testing, and supply of dentists (Table 4).

Among the more important indicators, the two indicators in level 1 (ie, blood sugar level and immunization and vaccination) were rated as significantly more important than the four indicators in level 3 (ie, HDL cholesterol, blood triglycerides, cancer screening detection, and total cholesterol). Therefore, based on the surveys and our analysis results, among these 13 agreeable health indicators, blood sugar level, and immunization and vaccination were the most important, and the perspectives of the participants were agreed upon across all three samples.

Furthermore, among the less important indicators, the indicator assigned to level 4 (ie, health literacy rate) was found to be significantly more important than the two indicators in level 6 (ie, self-rated health status and HIV testing) and the indicator assigned to level 7 (ie, supply of dentists). Additionally, the two indicators assigned to level 5 (ie, air quality index >100 and personal care needs) were found to be significantly more important than the indicator assigned to level 7 (ie, supply of dentists). Therefore, the survey and analysis results showed that the supply of dentists was the least important among the 13 health indicators, with agreed-upon perspectives across the three samples. Additionally, the inferential statistical test results among the levels provided more confidence in ranking the seven levels from the most important (ie, blood sugar level and immunization and vaccination) to the least important (ie, supply of dentists). The statistical significance test results among the levels provided evidence for prioritizing the 13 health indicators.

**Table 2:** The 13 indicators with nonsignificant mean differences across the three samples.

Health indicator and sources			Responses^a^, n (%)		Score^b^, mean (SD)		*P* value^c^
**Blood sugar level^d^**							
	ResearchMatch (n=694)	694 (100)		7.756 (1.6303)		.35	
	Clemson University (n=97)	97 (100)		7.588 (1.7485)			
**Blood triglycerides^d^**							
	ResearchMatch (n=694)	694 (100)		7.318 (1.7786)		.91	
	Clemson University (n=97)	97 (100)		7.34 (1.952)			
**HDL^e^ cholesterol^d^**							
	ResearchMatch (n=694)	694 (100)		7.307 (1.8264)		.53	
	Clemson University (n=97)	97 (100)		7.433 (1.9143)			
**LDL^f^ cholesterol^d^**							
	ResearchMatch (n=694)	694 (100)		7.43 (1.8489)		.53	
	Clemson University (n=97)	97 (100)		7.557 (1.9147)			
**Total cholesterol^d^**							
	ResearchMatch (n=694)	694 (100)		7.203 (2.0177)		.07	
	Clemson University (n=97)	97 (100)		7.598 (1.8465)			
**Personal care needs**							
	ResearchMatch (n=694)	694 (100)		6.816 (2.0786)		.14	
	Ohio University (n=362)	362 (100)		7.011 (2.3026)			
	Clemson University (n=97)	97 (100)		7.206 (2.1013)			
**HIV testing**							
	ResearchMatch (n=694)	694 (100)		6.616 (2.361)		.69	
	Ohio University (n=362)	362 (100)		6.619 (2.6439)			
	Clemson University (n=97)	97 (100)		6.835 (2.3747)			
**Self-rated health status**							
	ResearchMatch (n=694)	694 (100)		6.63 (2.2032)		.45	
	Ohio University (n=362)	362 (100)		6.619 (2.1504)			
	Clemson University (n=97)	97 (100)		6.918 (1.8912)			
**Supply of dentists**							
	ResearchMatch (n=694)	685 (98.7)		6.525 (2.0215)		.07	
	Ohio University (n=362)	362 (100)		6.34 (2.2572)			
	Clemson University (n=97)	97 (100)		6.041 (1.9944)			
**Health literacy rate**							
	ResearchMatch (n=694)	685 (98.7)		6.986 (2.0199)		.26	
	Ohio University (n=362)	362 (100)		7.061 (2.2617)			
	Clemson University (n=97)	97 (100)		7.34 (2.0098)			
**Immunization and vaccination**							
	ResearchMatch (n=694)	694 (100)		7.494 (2.1184)		.37	
	Ohio University (n=362)	362 (100)		7.666 (2.3041)			
	Clemson University (n=97)	97 (100)		7.722 (2.3968)			
**Cancer screening detection**							
	ResearchMatch (n=694)	694 (100)		7.217 (2.0625)		.52	
	Ohio University (n=362)	362 (100)		7.257 (2.3045)			
	Clemson University (n=97)	97 (100)		7.485 (2.0922)			
**Air quality index >100**							
	ResearchMatch (n=694)	685 (98.7)		6.736 (1.9232)		.78	
	Ohio University (n=362)	362 (100)		6.76 (2.125)			
	Clemson University (n=97)	97 (100)		6.887 (1.9304)			

^a^The independent variables were the three samples and the dependent variables were the individual survey responses.

^b^Items were rated on a scale of 0 to 10, where 0 refers to “not at all important” and 10 refers to “extremely important.”

^c^*P* values were based on analysis of variance or *t* test results for each health indicator among three samples; they are reported in the top row for each group.

^d^The Ohio University data set for this indicator was removed because a scale of 0 to 11 vs 0 to 10 was used.

^e^HDL: high-density lipoprotein.

^f^LDL: low-density lipoprotein.

**Table 3:** The seven levels of health indicators with no significant mean differences within levels.

Level and health indicators with no group mean differences^a^		Individual survey data^b^		
		Responses, n (%)	Score^c^, mean (SD)	*P* value^d^
**Level 1**				
	Blood sugar	791 (68.6)	7.74 (1.65)	.053
	Immunization and vaccination	1153 (100)	7.57 (2.20)	
**Level 2**				
	LDL^e^ cholesterol	791 (68.6)	7.45 (1.86)	N/A^f^
**Level 3**				
	HDL^g^ cholesterol	791 (68.6)	7.32 (1.84)	.77
	Blood triglycerides	791 (68.6)	7.32 (1.80)	
	Cancer screening detection	1153 (100)	7.25 (2.14)	
	Total cholesterol	791 (68.6)	7.25 (2.00)	
**Level 4**				
	Health literacy rate	1144 (99.2)	7.04 (2.10)	N/A
**Level 5**				
	Personal care needs	1153 (100)	6.91 (2.16)	.08
	Air quality index >100	1144 (99.2)	6.76 (1.99)	
**Level 6**				
	Self-rated health status	1153 (100)	6.65 (2.16)	.87
	HIV testing	1153 (100)	6.64 (2.45)	
**Level 7**				
	Supply of dentists	1144 (99.2)	6.43 (2.10)	N/A

^a^Independent variables were the indicators in each level.

^b^Dependent variables were the individual survey responses.

^c^Items were rated on a scale of 0 to 10, where 0 refers to “not at all important” and 10 refers to “extremely important.”

^d^*P* values indicate whether mean differences existed among the indicators within each level based on analysis of variance or *t* test results, and are reported in the top row of each group.

^e^LDL: low-density lipoprotein.

^f^N/A: not applicable; no comparison was conducted because the row has only one health indicator.

^g^HDL: high-density lipoprotein.

**Table 4:** Analysis of variance post hoc test results for the seven levels of indicators.

Health indicator level^a^	Health indicator level	*P* value
Level 1	Level 2	.58
Level 1	Level 3	<.001
Level 1	Level 6	<.001
Level 1	Level 7	<.001
Level 2	Level 4	.006
Level 2	Level 3	.68
Level 2	Level 5	<.001
Level 2	Level 7	<.001
Level 3	Level 4	.06
Level 3	Level 5	<.001
Level 4	Level 5	.27
Level 4	Level 1	<.001
Level 4	Level 6	<.001
Level 5	Level 6	.14
Level 5	Level 1	<.001
Level 5	Level 7	<.001
Level 6	Level 7	.28
Level 6	Level 2	<.001
Level 6	Level 3	<.001
Level 7	Level 2	<.001
Level 7	Level 3	<.001
Level 7	Level 4	<.001

^a^The independent variables were the levels of indicators and the dependent variables were the individual survey responses.

### Reliability of Survey Instruments

The 29 items from the survey instruments showed good levels of internal reliability (Cronbach α=.912), as did each of the three subsets related to institutions where the survey was administered (Table 5). Instruments with Cronbach α values equal to or higher than .7 are generally considered to be reliable [20].

**Table 5:** Reliability of the survey instrument.

Survey components and data analyzed			Cronbach α	
**Entire survey (all items)**				
	All three samples	.912		
	ResearchMatch	.922		
	Ohio University	.893		
	Clemson University	.925		
**Survey subscales**				
	Health risk and behavior indicators	.795		
	Health care	.613		
	Health care provider supply	.831		
	Blood tests in physical exams	.934		
	Other health indicators	.823		

## Discussion

### Principal Findings

Among all three samples, the ranking of the importance of 13 out of 29 (45%) health indicators showed agreement (Table 3). However, these health indicators were not necessarily more important than the other 16; instead, participants were observed to have perceived importance more consistently among these 13 health indicators. When we compared the 13 health indicators (Table 3) and their corresponding rankings in Table 1, we noticed that the 13 health indicators were placed between the 6th and 24th rankings in Table 1. This indicated more agreement among participants regarding the middle-ranked health indicators than the higher- or lower-ranked ones. The perspectives were more heterogeneous for the higher- or lower-ranked health indicators. Noticeably, the currently widely used standard individual health indicator, self-rated health status, was ranked 20th based on the results of the descriptive statistics. These results indicate a need for new and improved health indicators.

Among the 13 health indicators found in the seven levels, all levels were not significantly different from their immediate next level (Table 4); that is, there were no significant differences between levels 1 and 2 (ie, between n and n + 1). There were, however, significant differences between level 1 and levels 3 to 7 (ie, between n and any level higher than n + 1). These results pertain to the further prioritization of health indicators.

Given descriptive statistics and inferential test results, our findings among the 13 health indicators can reasonably be generalized to some extent to a broader population beyond our survey respondents. We do not claim the complete generalizability of our results mainly because our respondents were not perfectly representative of the composition of the American population. However, we believe that the 13 health indicators and their importance rankings within and among levels can provide substantial and useful evidence when such indicators need to be prioritized.

Cronbach α is one of the more cited statistics for informing internal consistency for the items of an instrument. If Cronbach α is greater than .7, the instrument is reliable [20]. The Cronbach α for the entire survey among the three samples was between .893 and .925. This indicates that we developed a reliable survey instrument. When examining the subscales, only the health care category, which included vaccination and immunization, insurance coverage, and personal care need, was below .7. The items in this category are among the most discussed topics in health care in the United States. Understandably, the reliability is lower since the respondents have relatively less consistent perspectives regarding these items.

### Significance and Comparison With Related Research

This study provides a more comprehensive understanding of the indicators affecting an individual’s health status, particularly as compared to self-rated health status, the most commonly used health status measurement [2]. Although there are advantages associated with using a single health indicator during clinical encounters, we believe that the multidimensional measurement of an individual’s health status may be more objective and can provide additional insights into the individual’s health status, particularly if we are concerned with improving and maximizing the preventive health care services offered. Obtaining these public perspectives is the first step toward a more accurate and effective measurement of individual health status.

This work can be potentially used in two ways: (1) to develop a more comprehensive and objective measurement of an individual’s health status and (2) to develop a health index for an individual. Additionally, these results can be used to prioritize various health indicators (eg, to distinguish between core and secondary indicators). They can also be referenced by designers and developers for EHR systems, personal health record (PHR) systems, or other data capture and analysis applications to determine what health indicators to include in the systems. Furthermore, these results can contribute to developing a health index, which can be used to stratify healthy research participants to make them more comparable. This would be analogous to the Charlson Comorbidity Index [21] or propensity scores [22], which are broadly used in clinical epidemiology data analytics, both of which, however, are disease oriented. Although the health indicators reported here are not in a formula format, this will be a focus for future research. These results set the foundation for further weighting, prioritizing, and validating health indicators via additional data resources.

Additionally, these measurements can track overall health status, measure the outcomes of preventive services, or aggregate data to examine community health. Although having more data points provides increased accuracy and specificity for health indicators embedded within an EHR or PHR, it is important to consider clinician burnout [23] when using technology. Therefore, it is necessary to be mindful of the impacts that creating more data capture requirements or expectations of clinical users may have. In this regard, prioritizing health indicators is a necessary step.

Over the years, other systems have been developed to assess various health risks and associations. The Johns Hopkins Adjusted Clinical Group system, developed and maintained by Johns Hopkins University for over 30 years, is a global tool used in population health analytics [24]. This system is focused on chronic conditions and comorbidities, and its goal is, therefore, fundamentally different from ours, which is to measure individual *health* status, versus *disease* status, more accurately. Another system, the Committee on Quality Measures for the Healthy People Leading Health Indicators [3], focuses more on quality measures with an aim to align the measurements within a framework of assessment, improvement, and accountability. The focus, however, is on monitoring and reporting at the population level, not necessarily individual health [3].

There are other health-related surveys broadly used worldwide. For example, the 36-Item Short Form Health Survey, developed by the RAND Corporation [25], measures quality-of-life and health outcomes. Similarly, compared with the related but smaller 12-Item Short Form Health Survey [26], our health indicators provide a more comprehensive measurement beyond physical and mental health. The 9-item Patient Health Questionnaire [27] is a validated tool that measures depression severity. However, we were looking for more objective indicators to measure an individual’s physical and mental health status in our work.

Our health indicators have good but not all-inclusive coverage. The Institute of Medicine’s Committee on the Recommended Social and Behavioral Domains and Measures for Electronic Health Records identified measures across the individual and neighborhood levels that involve sociodemographic, psychological, and behavioral data [4,5]. Among the 17 domains identified by the committee [4,5], 10 were included in our 29 health indicators. Healthy People 2030 [28] proposed 22 leading health indicators for different age groups, of which 16 are included in our health indicators.

### Limitations of This Study

The main limitation of this study is that it is only the first step in determining the importance of these health indicators and, notably, the results are subjective, as they are based on public perspectives. Further validation of these results via additional objective measures, such as health care expenditure by disease category [29] and the burden of illness estimates for specific disease categories [30], is needed to support these findings. In this study, for each health indicator, the sample size of valid responses ranged from 791 to 1153. We recognize that larger sample sizes may generate more conclusive and generalizable results. Therefore, our results about the 13 health indicators, even though they are inferential statistics, should be treated as preliminary baseline results; future research may be needed to validate these findings in other settings.

Another limitation concerns the survey respondents. Females comprised the majority of survey respondents, making up 72.1%, 77.7%, and 69.0% of the samples from Ohio University, ResearchMatch, and Clemson University, respectively. We noticed a similar phenomenon in other studies conducted via ResearchMatch. While we are pleased with the relatively large sample size, responses may reflect the perspectives of well-educated females more than those of other groups. For example, survey respondents with a college-level education and beyond represented 54.6%, 82.2%, and 74.0% of the respondents from Ohio University, ResearchMatch, and Clemson University, respectively.

In addition to the distribution imbalance in gender and educational background among our respondents, we also noticed that race and ethnicity groups (Multimedia Appendix 5) were not perfectly representative of the composition of the American population. The breakdown by racial groups among respondents of our surveys was as follows: White American, 87.3%; African American, 3.3%; Hispanic and Latino American, 2.2%; Asian American, 1.6%; Native American, 0.4%; and two or more races, 2%. We recognize that our data set’s gender and ethnicity imbalances are limitations of our current convenience sampling method. In the future, a stratified random-sampling method based on census-based population demographical data might provide more representative results and be a better option. This is a critical point that should be considered when using the results from this study.

### Future Research

We foresee several potential directions in which to continue this project. Our primary goal for future research is to validate the results obtained from the three completed surveys. This can be accomplished in several ways. Because we wish to measure individual health status accurately over time, the use of longitudinal data would be ideal. One data source is a citizen science project initiated by the National Institutes of Health, the All of Us [31] research program. Another source is the UK Biobank initiated in the United Kingdom [32], but the most ideal source would be well-documented longitudinal data of a group of individuals that include not only their EHR data but also other data that correlate with our health indicators. Such ideal data sources would allow for examining the corresponding health indicators and validation of the importance of health indicators via EHR records and additional health-related data. In this way, public perspectives will be considered along with more concrete quantitative evidence to ensure more confidence in prioritizing health indicators and using them for various purposes.

Additionally, to mitigate the effect of the current imbalances seen in respondents regarding gender, race and ethnicity, and other factors, we could explore the possibility of stratified random sampling to proactively select more representative participants. The respondent pool can be more proportionally representative of the composition of the American population. As a potential future project, we may also explore possible correlations between demographic variables and rating results.

### Conclusions

Well-designed health indicators are critical tools needed to accurately measure individual health status. They enable the determination of effective preventive services and verify their outcomes. Obtaining the public’s perspective on specific health indicators is the first step toward prioritizing them for analytical and clinical use. This study found that the top five–rated health indicators were drug and substance abuse, smoking and tobacco use, alcohol abuse, major depression, and diet and nutrition. Our respondents, however, had heterogeneous views on the top- and bottom-rated health indicators. The middle 13 health indicators were rated more homogeneously among all the respondents. These 13 health indicators were separated into seven levels based on their perceived importance, providing further evidence that was used to prioritize these health indicators. Levels 1 to 7 were organized based on the mean importance of health indicators from high to low within and between each level. Level 1 included blood sugar level and immunization and vaccination; level 2 included LDL cholesterol; level 3 included HDL cholesterol, blood triglycerides, cancer screening detection, and total cholesterol; level 4 included health literacy rate; level 5 included personal care needs and air quality index greater than 100; level 6 included self-rated health status and HIV testing; and level 7 included the supply of dentists. The results of this study can provide evidence to EHR or PHR system designers and developers, which they can then use to select health indicators to incorporate into their systems.

## Appendix

Multimedia Appendix 1 Survey instrument. Health indicators to measure an individual's health status: a public perspective survey.

Multimedia Appendix 2 Definition of the health indicators used in the survey.

Multimedia Appendix 3 Records from three data sets before and after data cleaning.

Multimedia Appendix 4 Survey codebook.

Multimedia Appendix 5 Demographic descriptive statistics for all respondents.

Multimedia Appendix 6 Total of nine indicators with homogenous variance.

Multimedia Appendix 7 Total of 20 indicators with heterogeneous variance.

Multimedia Appendix 8 Total of 16 indicators with significant mean differences: post hoc results for the three samples.

## Abbreviations

ANOVA: analysis of variance
EHR: electronic health record
HDL: high-density lipoprotein
LDL: low-density lipoprotein
PHR: personal health record

## Data Availability

This paper’s data sets and statistical analysis codes are available by request from the corresponding author.
