# Supplementary material for: Health Indicators as Measures of Individual Health Status and Their Public Perspectives: Cross-sectional Survey Study
Source: J Med Internet Res. 2022 Jun 21;24(6):e38099. doi: 10.2196/38099 (PMC9257608; doi:10.2196/38099)
Supplement: Multimedia Appendix 3 [file jmir_v24i6e38099_app3.pdf]

Multimedia Appendix 3. Records from three datasets: before and after data cleaning

	**Agreed and valid (based on validation question)**	**Incomplete records**	**Complete records**	**Invalid records (less than 3 mins)**	**Complete and valid records for analysis (after cleaning)**
Ohio University dataset	389	4	385	23	362
Clemson dataset	182	80	102	5	97
ResearchMatch	694	9	685	0	685
