# Supplementary material for: Health Indicators as Measures of Individual Health Status and Their Public Perspectives: Cross-sectional Survey Study
Source: J Med Internet Res. 2022 Jun 21;24(6):e38099. doi: 10.2196/38099 (PMC9257608; doi:10.2196/38099)
Supplement: Multimedia Appendix 4 [file jmir_v24i6e38099_app4.pdf]

Multimedia Appendix 4. Codebook

**Variables**	**Range**	**Notes for each code**
Age	[1-5]	1: < = 35<br>2: 36-45<br>3: 46-55<br>4: 56-65<br>5: > 65
Gender	[1-4]	1: Female<br>2: Male<br>3: Transsexual<br>4: Prefer not to answer
Professional group	[1-5]	1: Healthcare providers<br>2: Public health professional<br>3: Researcher who uses health indicator data<br>4: Other researchers<br>5: Other professional groups (specify)
Educational qualification	[1-5]	1: High school<br>2: Associate's degree<br>3: Bachelor's degree<br>4: Master's degree<br>5: Doctoral degree
Race	[1-7]	1: White American<br>2: African American<br>3: Hispanic and Latino American<br>4: Asian American<br>5: Native American (including Alaska

		native, native Hawaiian, and other<br>Pacific Islander)<br>6: Two or more races<br>7: Other
Sample	[1-3]	1: ResearchMatch<br>2: OU<br>3: Clemson
