# Supplementary material for: Health Indicators as Measures of Individual Health Status and Their Public Perspectives: Cross-sectional Survey Study
Source: J Med Internet Res. 2022 Jun 21;24(6):e38099. doi: 10.2196/38099 (PMC9257608; doi:10.2196/38099)
Supplement: Multimedia Appendix 5 [file jmir_v24i6e38099_app5.pdf]

Multimedia Appendix 5. Demographic descriptive statistics for all respondents

		Frequency (n)	Percent (%)
Source	ResearchMatch	694	60.2
	Ohio University	362	31.4
	Clemson University	97	8.4
	Total	1153	100
Age Group	< = 35	464	40.2
	36-45	163	14.1
	46-55	152	13.2
	56-65	201	17.4
	> 65	173	15
	Total	1153	100
Gender	Female	862	74.8
	Male	273	23.7
	Transgender	10	0.9
	Prefer not to answer	8	0.7
	Total	1153	100
Professional Group	Healthcare providers	232	20.1
	Public health professional	71	6.2
	Researcher who uses health indicator data	78	6.8
	Other researchers	204	17.7
	Other professional groups (Specify)	568	49.3
	Total	1153	100
Educational qualification	High school	218	18.9
	Associate degree	95	8.2
	College degree	367	31.8
	Master degree	327	28.4
	Doctoral degree	146	12.7
	Total	1153	100
Race	White American	1006	87.3
	African American	38	3.3
	Hispanic and Latino American	25	2.2
	Asian American	19	1.6
	Native American (Including Alaska native, native Hawaiian and other Pacific Islander)	5	0.4
	Two or more races	23	2

	Other	37	3.2
	Total	1153	100
