# Supplementary material for: Health Indicators as Measures of Individual Health Status and Their Public Perspectives: Cross-sectional Survey Study
Source: J Med Internet Res. 2022 Jun 21;24(6):e38099. doi: 10.2196/38099 (PMC9257608; doi:10.2196/38099)
Supplement: Multimedia Appendix 6 [file jmir_v24i6e38099_app6.pdf]

Multimedia Appendix 6. Total of 9 indicators with homogenous variance

**Health indicator**	**Levene test Sig.**
Blood sugar level	0.187
HDL cholesterol	0.223
LDL cholesterol	0.208
Total cholesterol	0.831
Immunization/vaccination	0.119
Insurance coverage	0.069
Cancer screening detection	0.053
Air quality index > 100	0.138
Self-rated health status	0.340
