# Supplementary material for: Health Indicators as Measures of Individual Health Status and Their Public Perspectives: Cross-sectional Survey Study
Source: J Med Internet Res. 2022 Jun 21;24(6):e38099. doi: 10.2196/38099 (PMC9257608; doi:10.2196/38099)
Supplement: Multimedia Appendix 7 [file jmir_v24i6e38099_app7.pdf]

Multimedia Appendix 7. Total of 20 indicators with heterogeneous variance

**Health indicator**	**Levenet Test Sig.**
Blood triglycerides	0.050
Alcohol abuse	0.004
Body mass index (BMI)	0.002
Diet and nutrition	0.001
Drug or substance abuse	0.000
Family history of cancer	0.046
Physical inactivity	0.000
Smoking, tobacco use	0.000
Sun protection	0.000
Personal care needs	0.048
Hypertension screening	0.001
HIV testing	0.026
High school diploma as a health indicator	0.000
Supply of dentists	0.032
Engagement in life	0.000
Health literacy rate	0.027
Major depression	0.001
Having a sense of purpose in one's life	0.000
Race and ethnicity	0.004
Unemployment	0.001
