# Supplementary material for: Health Indicators as Measures of Individual Health Status and Their Public Perspectives: Cross-sectional Survey Study
Source: J Med Internet Res. 2022 Jun 21;24(6):e38099. doi: 10.2196/38099 (PMC9257608; doi:10.2196/38099)
Supplement: Multimedia Appendix 8 [file jmir_v24i6e38099_app8.pdf]

Multimedia Appendix 8. Total of 16 indicators with significant mean differences post hoc results for the three samples

**Health indicator**			**Post hoc Sig.**
	ResearchMatch	Clemson	0.380
	OU	ResearchMatch	0.000
Alcohol abuse	Clemson	OU	0.052
	ResearchMatch	Clemson	0.698
	OU	ResearchMatch	0.000
Body mass index (BMI)	Clemson	OU	0.008
	ResearchMatch	Clemson	0.161
	OU	ResearchMatch	0.175
Diet and nutrition	Clemson	OU	0.016
	ResearchMatch	Clemson	0.100
	OU	ResearchMatch	0.000
Drug or substance abuse	Clemson	OU	0.498
	ResearchMatch	Clemson	0.006
	OU	ResearchMatch	0.000
Family history of cancer	Clemson	OU	0.884
	ResearchMatch	Clemson	0.540
	OU	ResearchMatch	0.000
Physical inactivity	Clemson	OU	0.450
	ResearchMatch	Clemson	0.004
	OU	ResearchMatch	0.000
Smoking, tobacco use	Clemson	OU	0.742

	ResearchMatch	Clemson	0.000
	OU	ResearchMatch	0.000
Sun protection	Clemson	OU	0.726
	ResearchMatch	Clemson	0.023
	OU	ResearchMatch	0.121
Insurance coverage	Clemson	OU	0.352
Hypertension screening	ResearchMatch	Clemson	0.750
	OU	ResearchMatch	0.007
	Clemson	OU	0.562
Engagement in life	ResearchMatch	Clemson	0.996
	OU	ResearchMatch	0.000
	Clemson	OU	0.000
Major depression	ResearchMatch	Clemson	0.941
	OU	ResearchMatch	0.022
	Clemson	OU	0.461
Having a sense of purpose in one’s life	ResearchMatch	Clemson	0.470
	OU	ResearchMatch	0.000
	Clemson	OU	0.000
Race and ethnicity	ResearchMatch	Clemson	0.659
	OU	ResearchMatch	0.000
	Clemson	OU	0.069
High school diploma	ResearchMatch	OU	0.000

	OU	Clemson	0.310
	Clemson	ResearchMatch	0.198
Unemployment	ResearchMatch	Clemson	0.898
	OU	ResearchMatch	0.003
	Clemson	OU	0.060

IV = the three samples, DV = Individual survey data.
